# Chronic Pain Management for Caregivers of Persons Living with Dementia

**DOI:** 10.1093/geroni/igaf122.3558

**Published:** 2025-12-31

**Authors:** Bianca Shieu, M Carrington Reid, Deami Cardenas, Glycel Vidaurri, Yousef Qan’ir, Min Wang, Lixin Song

**Affiliations:** University of Texas Health Science Center at San Antonio, San Antonio, Texas, United States; Weill Cornell Medicine, New York, New York, United States; UT Health San Antonio, San Antonio, Texas, United States; UT Health San Antonio, San Antonio, Texas, United States; UT Health San Antonio, San Antonio, Texas, United States; UTSA, San Antonio, Texas, United States; UT Health San Antonio, San Antonio, Texas, United States

## Abstract

Chronic pain is highly prevalent yet undertreated in persons living with dementia (PLWD), affecting up to 80% of PLWD and creating significant challenges for their family caregivers. The serious risks associated with pharmacological treatments like opioids necessitate safer, non-pharmacologic options. Auricular Point Acupressure (APA), a non-invasive and low-cost therapy, constitutes a promising alternative to mitigating pain in this expanding patient population. This study explored caregiver perspectives on using APA to inform the development of a caregiver-delivered training program. We conducted a qualitative descriptive study, guided by Bandura’s self-efficacy theory, with 22 primary family caregivers who provided care for PLWD between February and July 2025. Following IRB approval, semi-structured interviews were used to understand caregiver needs, interests, and preferred learning formats. Thematic analysis was performed using Atlas.ti V9. Caregivers expressed significant interest in learning APA, viewing it as a potentially valuable tool. They expressed a preference for a multi-faceted training program. Participants further requested a hybrid model that combines instructional videos for clear demonstrations with printed materials for quick reference. In addition, they identified a need for an introductory in-person workshop to become familiar with APA and an AI chatbot that would provide immediate answers to pressing questions. Participants felt that the training materials should focus on managing common conditions like low back, shoulder, and leg pain. These user-centered findings provide a foundational blueprint for developing a tailored APA training program that is designed to enhance family caregiver self-efficacy and provide a practical, safe strategy for pain management among PLWD.

